# Effects of Ellagic Acid Supplementation on Jejunal Morphology, Digestive Enzyme Activities, Antioxidant Capacity, and Microbiota in Mice

**DOI:** 10.3389/fmicb.2021.793576

**Published:** 2021-12-08

**Authors:** Qiuying Xu, Mingkang Shen, Yuxin Han, Hui Diao

**Affiliations:** ^1^Sichuan Nursing Vocational College, Chengdu, China; ^2^School of the 1st Clinical Medical Sciences, Wenzhou Medical University, Wenzhou, China; ^3^School of Nursing, Chengdu University of Traditional Chinese Medicine, Chengdu, China; ^4^Animal Breeding and Genetics Key Laboratory of Sichuan Province, Sichuan Academy of Animal Science, Chengdu, China

**Keywords:** ellagic acid, morphology, digestive enzyme activities, antioxidant, microbiota, Nrf2, HO-1

## Abstract

Ellagic acid (EA), a plant polyphenol mainly found in nuts and fruits, exhibits various biological effects. However, the effects of EA on intestinal health remain poorly understood. Hence, the present study aimed to assess the effects of EA supplementation on jejunal morphology, digestive enzyme activities, antioxidant capacity, and microbiota in C57BL/6J mice. A total of 144 mice were randomly assigned to three treatments groups: the control (CON) group received a standard pellet diet, the 0.1% EA group received a standard pellet diet plus 0.1% EA, and the 0.3% EA group received a standard pellet diet plus 0.3% EA. The mice were killed at the end of the experimental period, and jejunal samples were collected. The results revealed that the mice in the 0.3% EA group had higher (*P* < 0.05) average daily gain and greater (*P* < 0.05) jejunal villus height than those in the CON group. In addition, the jejunal lactase and sucrase activities were higher (*P* < 0.05) in the 0.1% EA and 0.3% EA groups, and the alkaline phosphatase activity was higher (*P* < 0.05) in the 0.3% EA group than in the CON group. Compared with the CON group, the administration of EA increased (*P* < 0.05) the superoxide dismutase and catalase activities but decreased (*P* < 0.05) the malonaldehyde content in the jejunum. Moreover, the jejunal messenger RNA expression levels of nuclear factor-E2-related factor 2 (*Nrf2*) and haem oxygenase-1 (*HO-1*) were higher (*P* < 0.05) in the 0.3% EA group than in the CON group. Furthermore, compared with the CON group, the count of *Escherichia coli* decreased (*P* < 0.05), and that of *Lactobacillus* species increased (*P* < 0.05) in the 0.3% EA group. In general, our findings indicate that the administration of EA can enhance the growth of mice, promote intestinal development, increase the antioxidant capacity, and regulate the intestinal microbiota.

## Introduction

The small intestine serves as the crucial site for nutrient digestion and absorption while simultaneously acting as an important line of defense against the invasion of antigens and pathogens (Nagler-Anderson, 2001; Hirata et al., 2007; Barszcz and Skomiał, 2011). However, the health status of the intestine in young animals could be easily deteriorated by many factors, including pathogen infection, inflammation, and oxidative stress (Liu, 2015; Wan et al., 2021); these factors lead to intestinal mucosal damage and dysfunction and, in turn, negatively affect the growth performance and health of animals (Liu et al., 2008). Interestingly, there is clear evidence that adequate nutrition can help maintain intestinal integrity in animals (He et al., 2017; Wan et al., 2018). Thus, it is extremely important to maintain the structural and functional integrity of the small intestine and to thereby ensure that its absorptive and protective functions are not compromised (Yu et al., 2010).

In recent years, there has been increasing interest in understanding the role of polyphenolic compounds and their possible mechanisms of action in maintaining intestinal health. Ellagic acid (EA), a natural phenolic phytochemical antioxidant, mainly occurs in vegetables and fruits, such as persimmon, raspberries, blackberries, and strawberries, in addition to nuts (Derosa et al., 2016). It has important biological activities, such as antioxidative (Yüce et al., 2007), anti-inflammatory (Marín et al., 2013), anticancer (Umesalma and Sudhandiran, 2011), and antidiabetic (Fatima et al., 2017) activities. Previous studies have shown that EA can scavenge free radicals (Zheng et al., 2020) and inhibit lipid peroxidation (Kilic et al., 2014). More recently, EA supplementation has been reported to improve the activity of antioxidant enzymes and ameliorate intestinal inflammation *in vivo* and *in vitro* (Han et al., 2006; Mishra and Vinayak, 2014). However, little information is available about the effects of different EA doses and their protective effects on intestinal health in mice. In light of the earlier information, we assessed the effects of EA supplementation on jejunal development and antioxidant capacity in mice.

## Materials and Methods

### Animals and Treatment

The Ethics Review Committee for Animal Experimentation of Sichuan Academy of Animal Science (Chengdu, China) approved the animal experimental protocols. The animal experimental protocols were conducted in accordance with the practical animal protection law and the Guide for the Care and Use of Laboratory Animals formulated by the National Research Council (China). A total of 144 C57BL/6J mice weighing 26–30 g were obtained from Dossy Experimental Animals Co., Ltd. (Chengdu, China) and randomly divided into three groups (each group has had 48 mice): the control (CON) group received a standard pellet diet, the 0.1% EA group received a standard pellet diet plus 0.1% EA, and the 0.3% EA group received a standard pellet diet plus 0.3% EA. All the mice were maintained at 25°C with a 12-h light/dark cycle and given food and water *ad libitum* for 21 consecutive days. All the mice were weighed on the morning of days 1 and 22 to calculate the average daily gain (ADG).

### Sample Collection

At the end of the experimental regimen and after 16 h of fasting, the mice were killed by ether anesthesia. The jejunum was removed, and the jejunal contents were collected and frozen at −80°C for microbial DNA analysis. Jejunal tissue samples were collected, washed in ice-cold physiological saline (0.9%), and blotted dry. Then, the jejunal section was divided into two pieces: one was fixed in 4% paraformaldehyde solution for histological studies, and the other was stored at −80°C for further analysis.

### Morphological Examination

The 4% paraformaldehyde solution-fixed jejunal samples were dehydrated in ethanol and embedded in paraffin. Then, 5-μm thick transverse sections were cut and stained with hematoxylin and eosin. The villus height and crypt depth of 10 well-oriented villi were measured using a BH2 Olympus microscope (Olympus, Tokyo, Japan).

### Jejunal Enzyme Activity Measurements

The jejunal mucosa was homogenized after ensuring that the weight of the intestinal mucosa (g)/volume of physiological saline pre-cooled on ice (ml) was 1:9 and was centrifuged at 2,500 × *g* for 10 min to obtain the supernatant. Then, the enzyme activities in the jejunal mucosal supernatant were measured strictly in accordance with the instructions provided in the respective kits (Nanjing Jiancheng Bioengineering Institute, Nanjing, China).

### Jejunal Antioxidant Capacity Measurements

The superoxide dismutase (SOD) activity, catalase (CAT) activity, malonaldehyde (MDA) content, and total antioxidant capacity (T-AOC) in the jejunal homogenates were measured using commercially available kits (Nanjing Jiancheng Bioengineering Institute, Nanjing, China).

### Messenger RNA Abundance Analysis

Total RNA was extracted from the jejunal mucosa using TRIzol reagent (Invitrogen, Carlsbad, CA, United States), according to the manufacturer’s instructions. The sequences of primers used in the present study are provided in Table 1. The RNA concentration was measured using NanoDrop 1000 (Thermo Fisher Scientific), and the RNA integrity was assessed by electrophoresis on 1% agarose gel. The reaction mixture consisted of 5-μl fresh SYBR^^®^^ Premix Ex Taq II (Tli RNase H Plus), 0.5-μl forward primer, 0.5-μl reverse primer, 1-μl complementary DNA, and 3-μl diethylpyrocarbonate-treated water. The polymerase chain reaction (PCR) protocol was as follows: 1 cycle at 95°C for 30 s, 40 cycles at 95°C for 5 s and 60°C for 34 s and 1 cycle at 95°C for 15 s, 60°C for 1 min and 95°C for 15 s. The relative expression levels of the target genes to the housekeeping gene (β-actin) were assessed using the 2^–ΔΔCt^ method (Che et al., 2017).

**TABLE 1:** Primer sequences of target and reference genes.

Genes	Primer sequence (5′–3′)	Product (bp)	GenBank accession
Nrf2	Forward: CCATGTGTGGCAGTCCATGAT Reverse: GCAGGCATACCATTGTGGAT	183	AH006764.2
HO-1	Forward: GAAATCATCCCTTGCACGCC Reverse: CCTGAGAGGTCACCCAGGTA	122	NM_010442.2
Keap1	Forward: GAGTAGAGGTAGGGGTCGCC Reverse: TCACGGTGACTAAGCACAGC	82	NM_016679.4
NQO1	Forward: CATTGCAGTGGTTTGGGGTG Reverse: TCTGGAAAGGACCGTTGTCG	111	NM_008706.5
β-actin	Forward: TGAGCTGCGTTTTACACCCT Reverse: GCCTTCACCGTTCCAGTTTT	198	NM_007393.5

*Nrf2, nuclear factor-E2-related factor 2; HO-1, heme oxygenase-1; Keap1, kelch-like epichlorohydrin-associated protein 1; NQO1, NADPH quinone acceptor oxidoreductase 1.*

### Bacterial DNA Extraction and Real-Time Quantitative Polymerase Chain Reaction

Approximately 1-g jejunal content was used for bacterial DNA extraction using the QIAamp DNA Stool Kit (Qiagen, Hilden, Germany) according to the manufacturer’s instructions. Bacterial DNA extracted from the jejunal contents was used for gene sequence amplification by quantitative PCR using the primers specified in Table 2. Primer specificity was assessed on the basis of the 16S rRNA gene sequence. The reaction conditions for quantitative PCR were as follows: 50°C for 2 min, 95°C for 5 min and 40 cycles of denaturation at 94°C for 20 s, primer annealing at a species-specific temperature for 30 s, and primer extension at 60°C for 1 min (Su et al., 2018).

**TABLE 2:** Primer sequences of target microbial populations in jejunal contents.

Items	Primer sequence (5′–3′)	Amplicon length (bp)
Bacteroidetes	Forward: GGARCATGTGGTTTAATTCGATGAT Reverse: AGCTGACGACAACCATGCAG	126
*Escherichia coli*	Forward: CATGCCGCGTGTATGAAGAA Reverse: CGGGTAACGTCAATGAGCAAA	95
Firmicutes	Forward: GGAGYATGTGGTTTAATTCGAAGCA Reverse: AGCTGACGACAACCATGCAC	126
*Lactobacillus*	Forward: AGCAGTAGGGAATCTTCCA Reverse: ATTCCACCGCTACACATG	345

### Statistical Analysis

All results are expressed as the means ± standard errors. Data were analyzed by one-way analysis of variance using the GLM procedure of SAS software (Version 9; SAS Institute, Inc., Cary, NC, United States). All statements of statistical significance are based on a probability of *P* < 0.05.

## Results

### Growth Performance

**FIGURE 1:**
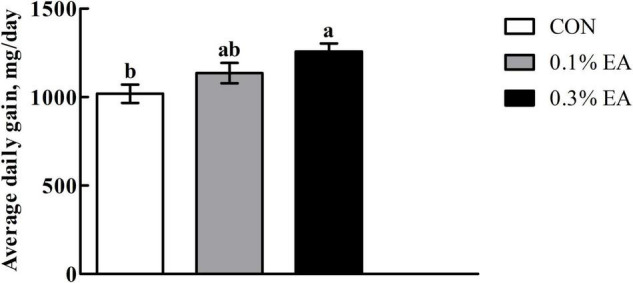
Effects of ellagic acid (EA) supplementation on growth performance of mice. Results are presented as means ± standard errors. *^a,b^*Mean values followed by different letters within a row indicate significant differences (*P* < 0.05). CON, control group, mice received a standard pellet diet; 0.1% EA, mice received a standard pellet diet plus 0.1% EA; 0.3% EA, mice received a standard pellet diet plus 0.3% EA.

As shown in Figure 1, the mice in the 0.3% EA group had significantly higher (*P* < 0.05) ADG than those in the CON group.

### Jejunal Morphology

**FIGURE 2:**
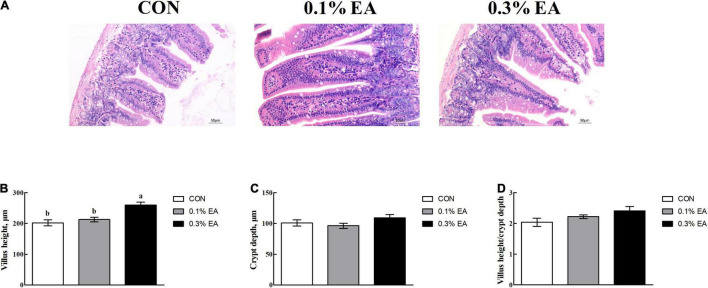
Effects of EA supplementation on jejunal morphology in mice. **(A)** Image showing jejunal morphology (200×); **(B)** villus height; **(C)** crypt depth; **(D)** villus height/crypt depth ratio. Results are presented as means ± standard errors. *^a,b^*Mean values followed by different letters within a row indicate significant differences (*P* < 0.05). CON, control group, mice received a standard pellet diet; 0.1% EA, mice received a standard pellet diet plus 0.1% EA; 0.3% EA, mice received a standard pellet diet plus 0.3% EA.

The jejunal villus height (Figure 2A) was greater in the mice fed with the EA diet than those fed with the basal diet. The villus height was greater (*P* < 0.05) in the 0.3% EA group than in the CON group (Figure 2B). However, the crypt depth (Figure 2C) and villus height/crypt depth ratio (Figure 2D) did not differ among the three groups (*P* > 0.05).

### Jejunal Enzyme Activities

The jejunal enzyme activities are presented in Table 3. Compared with the CON group, the jejunal lactase and sucrase activities were enhanced (*P* < 0.05) in the 0.1% EA and 0.3% EA groups. Moreover, 0.3% EA supplementation was found to increase (*P* < 0.05) the jejunal alkaline phosphatase activity.

**TABLE 3:** Effects of ellagic acid (EA) supplementation on jejunal digestive enzyme activities in mice.

Items	Treatment group*			*P*-value
	CON	0.1% EA	0.3% EA	
Lactase, U/mL	1.86 ± 0.19^c^	3.22 ± 0.25^b^	4.90 ± 0.37^a^	<0.001
Sucrase, U/mL	17.85 ± 0.51^b^	50.51 ± 0.83^a^	49.94 ± 1.39^a^	<0.001
Alkaline phosphatase, U/mL	4.65 ± 0.10^b^	9.75 ± 0.27^ab^	9.87 ± 0.21^a^	<0.001

*Results are presented as means ± standard errors.*

*^a–c^Mean values followed by different letters within a row indicate significant differences (P < 0.05).*

*CON, control group, mice received a standard pellet diet; 0.1% EA, mice received a standard pellet diet plus 0.1% EA; 0.3% EA, mice received a standard pellet diet plus 0.3% EA.

### Jejunal Microbiota

The counts of Bacteroidetes and Firmicutes species in the jejunal contents did not change significantly (*P* > 0.05) after EA supplementation (Table 4). Compared with the CON group, the count of *Escherichia coli* decreased (*P* < 0.05), and that of *Lactobacillus* species increased (*P* < 0.05) in the 0.3% EA group.

**TABLE 4:** Effects of EA supplementation on bacteria in jejunal digesta in mice [log 10 (copies per gram)].

Items	Treatment group*			*P*-value
	CON	0.1% EA	0.3% EA	
Bacteroidetes	7.14 ± 0.54	6.72 ± 0.45	6.41 ± 0.49	0.590
*Escherichia coli*	8.94 ± 0.36^a^	8.04 ± 0.29^a^	6.73 ± 0.40^b^	<0.001
Firmicutes	7.90 ± 0.44	7.62 ± 0.22	7.46 ± 0.26	0.620
*Lactobacillus*	4.39 ± 0.26^b^	4.75 ± 0.26^ab^	5.67 ± 0.44^a^	0.032

*Results are presented as means ± standard errors.*

*^a,b^Mean values followed by different letters within a row indicate significant differences (P < 0.05).*

*CON, control group, mice received a standard pellet diet; 0.1% EA, mice received a standard pellet diet plus 0.1% EA; 0.3% EA, mice received a standard pellet diet plus 0.3% EA.

### Jejunal Antioxidant Capacity

As shown in Table 5, EA supplementation increased (*P* < 0.05) the SOD and CAT activities and decreased (*P* < 0.05) the MDA content in the jejunum of mice.

**TABLE 5:** Effects of EA supplementation on jejunal antioxidant capacity in mice.

Items	Treatment group*			*P*-value
	CON	0.1% EA	0.3% EA	
SOD, U/ml	75.62 ± 7.71^b^	155.57 ± 9.83^a^	171.71 ± 9.53^a^	<0.001
CAT, U/ml	68.07 ± 2.00^c^	94.00 ± 2.24^b^	107.10 ± 2.44^a^	<0.001
T-AOC, U/ml	6.00 ± 0.98	6.52 ± 0.98	6.84 ± 0.33	0.768
MDA, nmol/ml	5.95 ± 0.36^a^	4.22 ± 0.16^b^	3.89 ± 0.12^b^	<0.001

*Results are presented as means ± standard errors.*

*^a–c^Mean values followed by different letters within a row indicate significant differences (P < 0.05).*

*CON, control group, mice received a standard pellet diet; 0.1% EA, mice received a standard pellet diet plus 0.1% EA; 0.3% EA, mice received a standard pellet diet plus 0.3% EA.

### Nuclear Factor-E2-Related Factor 2 Pathway-Related Gene Expressions Levels

**FIGURE 3:**
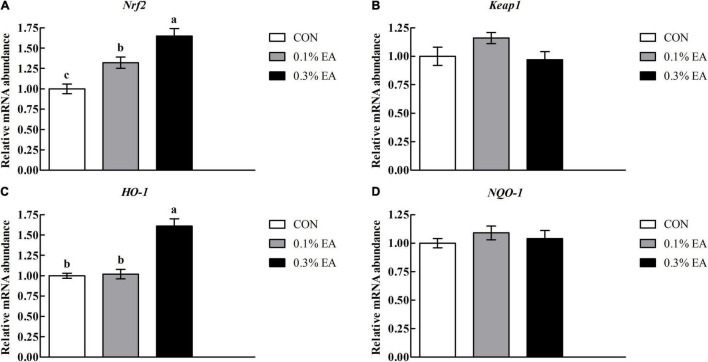
Effects of EA supplementation on nuclear factor-E2-related factor 2 (Nrf2) pathway-related mRNA expression levels in mouse jejunum. **(A)** Nrf2; **(B)** Keap1; **(C)** HO-1; **(D)** NQO-1. Results are presented as means ± standard errors.^*a*–*c*^Mean values followed by different letters within a row indicate significant differences (*P* < 0.05). CON, control group, mice received a standard pellet diet; 0.1% EA, mice received a standard pellet diet plus 0.1% EA; 0.3% EA, mice received a standard pellet diet plus 0.3% EA.

The differences in jejunal nuclear factor-E2-related factor 2 (Nrf2) pathway-related gene expression levels among the three groups are presented in Figure 3. The jejunal messenger RNA (mRNA) expression levels of *Nrf2* and haem oxygenase-1 (*HO-1*) were higher in the 0.3% EA group (*P* < 0.05) than in the CON group. However, no significant differences (*P* > 0.05) were noted in the mRNA expression levels of *Keap1* and *NQO-1* among the three groups.

## Discussion

The intestine has an enormous surface area that is optimized to efficiently absorb nutrients, water, and electrolytes from food (König et al., 2016). Maintaining the normal morphology of the intestinal mucosa is essential to ensure efficient digestion and absorption in animals (Mou et al., 2019). The villus height, crypt depth, and villus height/crypt depth ratio are the most direct indices that can reflect the morphology of the small intestine (Qin et al., 2018). In the present study, the jejunal villus height was found to be greater in the 0.3% EA group than in the CON group. This finding was consistent with that of Sun et al. (2017), suggesting that EA administration can change the morphological structure of the mouse jejunum and can promote the development of the small intestine in mice. It is well known that digestive brush border enzymes mainly attach to the apical parts of villi (Asp et al., 1975). Moreover, the proliferation of villi helps more digestive enzymes to enter the intestine, thereby facilitating the digestion and absorption of more nutrients (Cao et al., 2015). However, changes in intestinal morphology are associated with alterations in enzyme activities (Wan et al., 2017). In the current study, we found that 0.1 or 0.3% EA supplementation enhanced the jejunal lactase, sucrase, and alkaline phosphatase activities in mice. BW changes are a comprehensive reflection of health. In our study, mice fed the 0.3% EA diet had higher ADG than those fed the basal diet. This is a comprehensive reflection of EA-induced small intestinal development and improved intestinal nutrient digestion and absorption capacity.

The intestinal antioxidant capacity is also crucial for maintaining intestinal health (Kim et al., 2021). The body has a series of defense mechanisms for controlling oxidative stress; one of these is the enzymatic antioxidant system (Pari and Sivasankari, 2008). SOD and CAT are important enzymatic antioxidants that can provide major antioxidant defenses against ROS (Bhattacharyya et al., 2014; Shen et al., 2014). In the present study, the jejunal SOD and CAT activities significantly increased, whereas the jejunal MDA content decreased in mice fed EA. These findings suggest that EA can improve the jejunal antioxidant capacity. Evidence suggests that the antioxidant effect of EA may be mediated by the stimulation of Nrf2 and the activation of antioxidant response elements, such as HO-1 (Ding et al., 2014). Nrf2 is an important transcription factor that can control the induction of antioxidant genes (Baluchnejadmojarad et al., 2017). In this study, we found that EA supplementation increased Nrf2 and HO-1 mRNA expression levels in the mouse jejunum, suggesting that EA can protect against oxidative stress *via* the Nrf2/HO-1 pathway.

The intestinal microflora plays an important role in maintaining intestinal microenvironment homeostasis (Liu et al., 2019). Previous studies have revealed an increase in the abundance of Firmicutes and *Lactobacillus* species and a decrease in the abundance of Verrucomicrobia and Bacteroidetes species in mice after polyphenols supplementation (Kim et al., 2021; Lu et al., 2021). Our results demonstrated that EA supplementation decreased the count of *E. coli* but increased the count of *Lactobacillus* species. These results may be attributed to the bacteriostatic or bactericidal action of polyphenols or their ability to inhibit the adhesion of pathogenic bacteria to the intestinal tract cells (Abu Hafsa and Ibrahim, 2018). Furthermore, the intestinal microflora has been reported to affect intestinal morphology (Forder et al., 2007). For instance, dietary *Lactobacillus casei* supplementation was found to increase the count of *Enterobacteriaceae* species and improve the intestinal villus length in mice (Allori et al., 2000). In the present study, the increased villus height noted in the 0.3% EA group was consistent with the increased *Lactobacillus* count in the jejunum, similar to previous findings.

## Conclusion

In conclusion, the present study demonstrated that EA administration could enhance the growth of mice, promote jejunal development, improve the jejunal antioxidant capacity, and regulate the intestinal microbiota. These findings provide guidance on EA supplementation.

## Data Availability Statement

The original contributions presented in the study are included in the article/supplementary material, further inquiries can be directed to the corresponding author.

## Ethics Statement

The animal study was reviewed and approved by the animal experimental protocols were approved by the Ethics Review Committee for Animal Experimentation of Sichuan Academy of Animal Science (Chengdu, China). The animal experimental protocols were conducted in accordance with the practical animal protection law and the Guide for the Care and Use of Laboratory Animals formulated by the National Research Council (China).

## Author Contributions

QX and HD conceived the present study and wrote the manuscript. MS and YH conducted the experiments and performed the data analysis. All authors contributed to the article and approved the submitted version.

## Conflict of Interest

The authors declare that the research was conducted in the absence of any commercial or financial relationships that could be construed as a potential conflict of interest.

## Publisher’s Note

All claims expressed in this article are solely those of the authors and do not necessarily represent those of their affiliated organizations, or those of the publisher, the editors and the reviewers. Any product that may be evaluated in this article, or claim that may be made by its manufacturer, is not guaranteed or endorsed by the publisher.
